# Editorial: Targeting gut microbiota modulation by dietary supplementation to improve metabolic diseases

**DOI:** 10.3389/fmicb.2023.1260520

**Published:** 2023-08-14

**Authors:** Lili Sheng, Xiaojiao Zheng, Lin Shi, Prasant Kumar Jena

**Affiliations:** ^1^School of Pharmacy, Shanghai University of Traditional Chinese Medicine, Shanghai, China; ^2^Center for Translational Medicine, Shanghai Sixth People's Hospital Affiliated to Shanghai Jiao Tong University School of Medicine, Shanghai, China; ^3^College of Food Engineering and Nutritional Science, Shaanxi Normal University, Xi'an, China; ^4^Department Pediatrics, Cedars-Sinai Medical Center, Los Angeles, CA, United States

**Keywords:** diet, gut microbiota, NAFLD, metabolism, probiotic

Human gut microbiota is a complex and dynamic ecosystem, which is colonized by trillions of microorganisms (de Vos et al., 2022). Gut microbiota contributes to various physiological processes, such as immune maturation and homeostasis, protecting against pathogen overgrowth, regulating intestinal endocrine functions and neurologic signaling, modulating energy metabolism, and producing functional metabolites (Cani et al., 2019). Emerging evidence shows an altered gut microbiota composition and function in patients with various health issues including metabolic diseases (Wu et al., 2021). Animal studies further confirmed a causal role of gut microbiota in metabolic disorders, such as non-alcoholic fatty liver disease (NAFLD), obesity, and type 2 diabetes mellitus (T2DM) (Sung et al., 2017; Li et al., 2023). Diet is a key modifiable factor affecting the composition and function of the gut microbiota. Thus, gut microbiota-targeted therapies, such as diet supplementation with nutrients, probiotics, prebiotics, and natural compounds, might be a promising approach to counteract the dysbiosis-related deleterious consequences.

In this Research Topic, recent findings in the modulation of gut microbiota for alleviating NAFLD as well as the compositional and functional changes of gut microbiota during the improvement of certain health issues are highlighted. It comprised six studies: the roles of vitamin D (VD) (Zhang et al.) and *Lactobacillus plantarum* in alleviating NAFLD via regulating gut microbiota (Wen et al.), the effect of Roux-en-Y gastric bypass (RYGB) for weight loss on modulating gut microbiota (Yang et al.), and the effect of prebiotics on the resilience of infant gut microbiota to amoxicillin/clavulanate perturbation (Endika et al.) were explored. In addition, the factor that affects seasonal differences in the intestinal flora was reported (Li et al.), and the effects of dietary and microbial nutrients on improving premature ovarian insufficiency (POI) were reviewed (Han et al.).

The study conducted by Zhang et al. aimed to investigate the effect of vitamin D on alleviating HFD-induced NAFLD. The study found that VD intake alleviated the HFD-induced NAFLD features and liver injury and reversed the HFD-decreased abundance of *Porphyromonadaceae_unclassified* and *Prevotella* genus, while boosting the abundance of *Lactobacillus* genus. Metabolomics data revealed that VD intake increased tyrosine metabolism, tryptophan metabolism, arginine biosynthesis, and sphingolipid metabolism. Integrated analysis of the gut microbiota and metabolism suggested that genus *Prevotella* positively correlated with tryptophan metabolism and sphingolipid metabolism as well as certain metabolites such as serotonin, melatonin, tryptamine, L-arginine, and 3-dehydrosphinganine. This comprehensive integrated microbiota and metabolomic analysis demonstrated that VD supplementation could be a potential intervention for anti-NAFLD by targeting the specific microbiota and metabolism.

Probiotic *Lactobacillus plantarum* ATCC14917 has been reported to alleviate the progression of atherosclerotic lesion formation and improve inflammation and oxidative stress in mice (Hassan et al., 2020). However, whether *L. plantarum* ATCC14917 could ameliorate NAFLD has not been elucidated. In this study, Wen et al. found that both low and high doses of *L. plantarum* ATCC14917 could alleviate NAFLD features and improve serum lipid metabolism as well as regulate gut microbial composition and structure. Additionally, the activity of superoxide dismutase and the content of GSH-Px were increased, but malondialdehyde content was reduced in the liver compared with the HFD group. With *L. plantarum* ATCC14917 intervention, the levels of these inflammatory cytokines were significantly reversed in the treatment groups, accompanied by an altered TLR4/NF-κB signaling pathway. These results revealed that *L. plantarum* ATCC14917 can be an alternative therapy for the intervention of NAFLD.

Obesity has become a global health and socioeconomic problem, while Roux-en-Y gastric bypass (RYGB) and sleeve gastrectomy (SG) are the two most used strategies for weight loss. In this study, Yang et al. performed a comprehensive analysis and found that SG surgery induced modest microbial alteration compared to RYGB. RYGB operation significantly decreased Firmicutes/Bacteroides (F/B) ratio and increased the proportion of *Escherichia, Bacteroides*, and *Akkermansia* genera, which might suppress the sphingosine and phytosphingosine metabolisms. Overall, the study revealed that the beneficial effect of RYGB in weight loss might be through the regulation of bacterial–metabolite crosstalk.

Dietary-dependent recovery of host metabolism from antibiotic exposure has been explored previously (Li et al., 2021). Endika et al. investigated two prebiotics, namely 2′-fucosyllactose (2′-FL) and galacto-oligosaccharides (GOS), on the recovery of 1- and 3-month-old infant gut microbiota to amoxicillin-/clavulanate-induced changes in microbiota composition and activity. In the *in vitro* colon model, amoxicillin/clavulanate time dependently caused deviations in microbiota composition. Supplementation of 2′-FL in the colon model of 1-month-old infant gut microbiota promoted the recovery of microbiota with mixed taxa and restored the production of propionate and butyrate. However, GOS supplementation in the colon model using the 3-month-old infant gut microbiota promoted the recovery of *Bifidobacteria*, dominated microbiota, and increased the production of acetate and butyrate. These findings suggested that prebiotics, such as 2′-FL and GOS supplementation, could have added value in promoting the recovery of microbiota in the gut of antibiotic-treated infants.

Seasonal differences in the gut microbiota composition have been reported (Huang and Liao, 2021; Marsh et al., 2022). However, the mechanism underlying these differences remains unclear. Li et al. analyzed the gut microbiota changes and intestinal water metabolism under four seasons that were simulated using the balanced temperature and humidity control system and found that seasonal changes could affect the concentration of colonic 5-hydroxytryptamine and vasoactive intestinal peptide in rats, accompanied by altered contents of AQPs through cAMP/PKA pathway resulting in the alteration of the intestinal water metabolism. These results uncovered the mechanism of how seasonal factors affect the level of intestinal water metabolism that leads to seasonal differences in gut microbiota.

Nutritional and dietary supplements are getting more and more attention for their health effect in regulating lipid and glucose metabolism. In addition to metabolic regulation, Han et al. summarized dietary and microbial nutrients as well as their roles and applications for prolonging reproductive lifespan in female patients. This review discussed the effects and mechanisms of dietary nutrients and microbe-related nutritional substances (including carbohydrates, fat and lipoprotein, protein and polypeptide, vitamins, phytoestrogens, probiotics, prebiotics, and synbiotics) on improving premature ovarian insufficiency, providing important information for the better and healthier life of female individuals.

Overall, these studies provide important insights into the effects of various dietary nutrients, probiotics, and prebiotics on host health by regulating gut microbiota composition and function as well as metabolites. However, further research is needed to fully understand the causal relationship between health improvement and gut microbiota changes upon dietary intervention. Additionally, germ-free mice or fecal microbiota transplantation should be considered to explore the role of gut microbiota in regulating host metabolism after dietary or microbial metabolites intervention. Together, these studies contribute to our understanding of dietary supplementation on host health through gut microbiota and provide important insights into the study of their beneficial effect mechanism.

## Author contributions

LShe: Writing—original draft, Writing—review and editing. XZ: Writing—review and editing. LShi: Writing—review and editing. PJ: Writing—review and editing.
